# Miscellanies

**Published:** 1840-01-01

**Authors:** 


### MISCELLANIES.

### ITINERANT PHRENOLOGISTS.

Phrenology appears to be in danger from a bastard brood of her own, who ape the mountebank's trade, and sell characters in the most approved fortune-telling fashion. These gentry have quitted the lines upon the palm, and have taken to the bumps upon the head. What sort of market they are making of them let the Phrenological Journal tell.

" Their advertisements of advice and manipulation are now frequently seen in the London papers; and some of them make a regular (or rather an irregular) profession of itinerating from town to town to sell manipulations. These latter usually premise a course of phrenological lectures, 'first lecture gratis,' to advertise their skill and custom. They also procure eulogistic paragraphs in the newspapers read in the place, by disbursement of a proportionate share of the profits of their trade, which easily procures for them these paragraphs to any extent of eulogy. A host of persons have their curiosity aroused by these arts, and make haste to purchase the advice and manipulations of the phrenological mountebank. Each who gets his own head manipulated is certain to carry his paper-voucher to some of his acquaintances, and to recommend them to follow his example. Herein lies the 'whole art and mystery' of the profession. People must be pleased, and must be made to talk and recommend. The ready method of insuring these results, is to seize a few salient points of character, clearly shown on the head, which is an easy enough task to any one having only a very slight knowledge of Phrenology; and the rest of the sketch is filled up with flatteries, or is penned in terms of sufficient ambiguity for admitting of a construction acceptable to the vanity and self-pride of the party manipulated. The acquaintances of the poor egotist laugh in their sleeve at his credulous vanity, but of course gratify him by declaring the portrait to be a wonderful likeness, and he is fully and instantly convinced of 'the truth of Phrenology;' becoming also an especial trumpeter of the skill of the manipulator. This creditable mode of earning a living out of the ignorance and vanity of others is not confined to the male sex. We have seen advertisements of lectures on Phrenology by 'ladies,' who also manipulate, and give advice to young men touching marriage and other interesting subjects."

Of course, there cannot be a doubt that this sort of humbug is injurious to phrenology. It may cause the unthinking to laugh, but, amongst phrenologists, it cannot but make " the judicious grieve." The *practice* of phrenology is fraught with danger, and requires the greatest circumspection. If ignorant and unprincipled quacks make it an engine of downright imposture, all that is philosophical in it will be damaged in the world's esteem.

The editor of the Phrenological Journal goes on to say :—

" But the art is itself too profitable to be suppressed by the discountenance of the superior phrenologists. They might as well attempt to stop the sale of quack medicines, as to suppress the sale of phrenological flatteries. They may refuse to countenance or encourage itinerant dealers, which of course all reputable phrenologists will do; but the ignorant and the joke-loving, the credulous and the curious, will still flock to the mountebanks; and thus the disadvantages will continue to the uttermost, without the counterbalancing advantages. To ensure the latter, we think it would be expedient for the superior phrenologists to abstain from denouncing the custom *in toto;* since the more it is condemned by them, the lower will be the grade of those who do follow it; by wholesale condemnation, it will be forced downwards instead of upwards. But if it must and will be followed, of which we think there can be no doubt, it were better raised to dignity than depressed into degradation.

Who, then, should be the parties encouraged to practise the art? The subject is not yet sufficiently advanced, to constitute a profession by itself; though a succeeding generation may expect to see this done. Of the professions now followed, Phrenology is clearly the most intimately connected with that of medicine; and to the medical profession accordingly we should gladly see it resigned. Their professional education, their habits of life, and the friendly intimacy so frequently subsisting between families and their medical advisers, are all in favour of this; and if a few personally respectable and *settled* medical practitioners would allow themselves to be consulted phrenologically as well as medically, the chief objections to the custom would speedily disappear, and all the more intelligent and respectable portion of the public would learn to see the quacks and itinerants in their true colours. We now speak of the medical profession collectively; many individuals of it would prove incompetent to practise Phrenology, through lack of general attainments."

We are afraid that these manipulators will make phrenology look foolish,

---

### A great Operator and his Operations.*

The following is a sketch of a great operator on the Continent. It is evidently from the life.

"On June 26th last, I paid a visit to his clinical hospital, and saw him for the first time. He is somewhat above the middle height, and has a fine muscular development, especially of the arm and leg, to which he probably owes much of his dexterity. His crown is bald, hair inclining to grey, nose Roman, and whiskers worn across the cheek. He came into the theatre to perform two operations, dressed in a short linen jacket, reaching not quite to the hips, and with sleeves terminating above the elbow; the forearm was bare, and he had an oil-cloth apron tied round him."

This gentleman, something between an hostler and a knacker, was the celebrated Langenbeck. The amputation he performed was done thus:—

"The patient was a youth, with scrofulous disease of the knee-joint. The preparations for the operation were exceedingly simple. Two basins with water and sponges on the floor; a few ligatures in the hand of one assistant, a saw, and three or four pairs of Amussat's and the common forceps, in the hands of others; a case of scalpels, and a case containing two amputating knives on a little side-table. The simplicity of the apparatus contrasted strongly with the pails and saw-dust, and glittering array of knives and scissors, and scalpels in extended file, flanked by tourniquets, bandages, strips of plaster, piles of rag, &c. witnessed in some English hospitals. The knife which Langenbeck uses is supposed to be peculiar to him, and in consequence has received his name; but its prototype may be seen in the hands of any butcher in England, being a short knife, rounded at the point. The blade and handle are each about four inches long; the former about 5-8ths in breadth. The artery being compressed by an assistant, Langenbeck took the knife just described in his hand, stepped back two steps—stood in a theatrical attitude, with the knife elevated above his head; then darted upon his patient, and made a deep gash on the outer part of the lower third of the thigh, cutting half away through to the bone; the knife describing a slight curve downwards and backwards; another application of the knife, slanting upwards, reached the bone. The same method was followed on the

---

* Med. Gaz. Aug. 24, 1839.

opposite side. The knife was then made to describe one or two turns round the bone; was changed for the saw; the muscles were retracted by the hand, and in about one minute from the commencement of the operation the limb was severed. The artery was sufficiently compressed to prevent a jet, but not enough to secure the patient from the loss of more blood than he ought to have suffered, considering his age and great debility. After tying the arteries, the thigh was rolled, and the face of the stump slightly covered with a piece of rag. The patient was then taken to his bed, and rag, wet with cold water, applied. In an hour or two, the lips of the flap were united by four or five sutures. Next day I found the roller removed, and nothing upon the stump except a strip of wet paper along the line of union. This mode of water-dressing has been practised by Professor Langenbeck for about thirty years. It is not so good as Liston's, because there is nothing to prevent the paper drying. To remedy this, a painter's brush and a basin of water stand close to the patient's bed, that the paper may be moistened from time to time."

Here are some of the faults and all the advantages of a Continental operation. The disregard of the loss of blood, and the theatrical display are unquestionably faults—the simplicity, boldness, celerity, and dexterity as unquestionably advantages. Of the dexterity it may be said, as Gibbon said of personal beauty, that those who despise, are those who have not got it.

*A Clinique.*—" Next came a boy with a tumor in the upper part of the thigh. It was a doubtful case, and there was a long inquisition upon it. It is well known, that in the German cliniques there is a student in charge of each patient, who is examined by the professor, respecting the case, in the presence of the class. The questions put are those suggested by the anatomical and physiological relations of the part affected; and the professor rectifies, explains, or illustrates, the answers: in doing which, Langenbeck mingles a little wit, a good deal of theatrical gesticulation, and a good deal of extraneous matter, with much useful information. The wit, gesticulation, and foreign matter, are far from being useless, as they serve to impress the case upon the memory of the students, by exciting and keeping up their attention. Indeed, nothing is more dull and soporiferous than one of these cliniques conducted with legitimate professional gravity. Langenbeck is generally censured for his declamatory and showy manner, in which he strongly resembles Lisfranc; but he is in the right."

We believe that it will be admitted that the mass of medical students are not philosophers. The most effective lecturers and most successful teachers have found, we fancy, that a little *piquance* is necessary to make the dull matter go down. Science is well for the scientific, but the young gentlemen of a class like a little dash of fun in it. And provided that the science is not lost, the fun is not so reprehensible.

---

### FRIGHTFUL STATE OF THE CLERGY.

The passage we are about to quote is really alarming. The Church *is* in danger now. We would advise all parsons of the good old style to beware of their evangelical brothers. Better stick to the "jolly full bottle," than join the snivelling tea-totallers.

It appears that in New England the clergy are in such a strait, that they have set a new quinsy a going. This is called the "*clergyman's sore-throat,*" and is distressingly prevalent. A writer in the Boston Journal gives a harrowing account of the cloth.

" Half a century ago, most of our clergymen laboured some, particularly in the time of getting hay. Nearly all made their gardens and cut their wood; and several of them did considerable towards cultivating their own small farms. Their parochial duties were comparatively light; they seldom preaching more than two sermons a week; and from the composition of these they were frequently relieved by exchanges about once a month, or oftener during Summer. Most of them smoked tobacco, and some of them chewed. I recollect but one old minister who took snuff. They all drank cider daily, and when fatigued, generally took some flip or toddy. Very few of them, if any, however, were in the habit of drinking ardent spirit daily. That refreshing drink, small beer, made from malt, was considered nearly as necessary as bread in a family. Thus they frequently led very quiet and regular lives, without being exposed to intense excitement of mind, or over-fatigue of body. On the whole, they were probably as healthy, and lived as long, as most other classes of the community. My own father lacked only about two months of being eighty-eight years old, when he died.

Within the last twenty years, it would seem that clergymen, in about equal proportions, in our different denominations, have been breaking down by scores, and more particularly as respects their voice. I have been more familiar with these cases among the Methodists, Episcopalians, and Congregationalists, though I presume there is a proportional number among the Baptists.

There have been some very striking changes in the condition of most of the clergy in New England since the commencement of the present century. In the first place, their duties are much increased, public opinion requiring of them, in most places, much greater exertions than their predecessors commonly made. The religious meetings, benevolent associations, societies for promoting various objects, and new organizations of almost every description, are, perhaps, ten times as numerous as they were forty years ago. To say that four times as many sermons and addresses are now delivered before a given population, as was formerly the case, would be an estimate, probably, much below the fact. Besides these additional labours, the habits and manner of living of very many clergymen, are essentially changed. Few now have either leisure or land to enable them to attend to agriculture. Most of them have renounced tobacco. When they are fatigued, the slow process of self-restoration is adopted, instead of the expedient of more suddenly reviving the exhausted powers of the system, by the diffusible stimuli. Alcohol in all its forms is proscribed. Few use wine, and most even dispense with cider. Many families have dropped the use of coffee, some of tea, and a considerable number have materially lessened the quantity of animal food. Malt beer, that ancient, harmless beverage, is scarcely known."

We have decidedly no fancy for New England. Think of undergoing eight sermons a week, and trusting to the "slow process of self-restoration" for a recovery! No constitution could stand it.

Seriously, is it not something worse than laughable to see what religious enthusiasm leads to? It breaks down the body, wears out the mind, and affecting to render men wiser, happier, and better, it proscribes the bounties of Nature, and denounces that disposition to innocent enjoyment which a benevolent Creator has blessed us with. And what are the consequences? Physical infirmity, mental delusion, the gloomy home, the fanatic meeting, the bed of sickness, and the madhouse.

---

### Dislocation of the Head of the Radius Backwards.

Sir, Andover, Oct. 20th, 1839.

My attention has recently been called to the details of a case, in the 30th Vol. of the Medico-Chirurgical Review, of Dislocation of the Head of the Radius backward; which is justly commented upon as being exceedingly uncommon.

As every mite of information becomes valuable when disseminated in association with facts of the same class, I am induced to trouble you with the following similar case which came under my care some time since.

On the 10th of September, 1838, I was met (at some distance from the town), by a boy about twelve years of age, who requested me to examine his left arm, which he said he thought was broken in a fall.

Upon doing so I found the head of the radius projecting backwards, and so fixed as to render the fore-arm nearly motionless. Having no one to assist me; by grasping the elbow with the left hand, pressing with the fingers firmly upon the head of the displaced bone, and, with the right, firmly and steadily supinating the hand, making at the same time moderate extension, the dislocation was immediately and easily reduced. The accident was followed by but little inflammation, which readily subsided in the course of a few days, by the use of evaporating lotions, &c.

The reduction in this case was, no doubt, the more readily effected, in consequence of the youth of the patient, and the shortness of the time which had elapsed since the accident, being not more than a few minutes.

I am, Sir,

Your obedient servant,

OCTAVIUS HAMMOND.

To the Editor of the
Medico-Chirurgical Review.

---

### SUPPLY OF SUBJECTS FOR DISSECTION.

He must be a facetious man who can talk of the present supply of subjects. It is an algebraical quantity—something minus nothing. The Anatomical Bill has worked, or has been worked charmingly. Each year since its introduction the number of subjects has decreased, until it has reached what we may hope to be a minimum.

The Inspector of Anatomy has been for some time a procurator of bodies, and a distributor of them. For this he is paid pretty handsomely. What have been the fruits of his procuratorship, let the present dearth testify—what the consequences of his distributorship, it is not so easy to divine. The system is radically bad which gives him either office. A government functionary is the worst person in the world to send to a parish board. He wears the odious garb of authority, and parish patriotism is in arms against him. If this be true of government functionaries in general, how true it must be of the existing one in particular. The universally expressed opinion of the teachers, their vote of censure, the representations to the Home-office, and the fruits of Dr. Somerville's labours, speak a language which cannot be misunderstood, and ought not to be disregarded by the government. Let the Inspector be what the Act intended—an inspector and no more. Dr. Somerville must be squeezed to his original proportions.

The business has become so tangled that it is difficult to know what to do. But the best plan is to appoint the College of Surgeons to the post of obtaining and of distributing bodies. It is a farce to talk of the teachers managing that matter themselves. They have tried and failed. Then who are to do it? We know of none so adapted to the office as the Council of the College. Some parties affect to dread abuse and jobbing. But we would observe that jobbing and abuses and intrigues may be found amongst anatomical teachers, as well as in the College, and publicity is the great safeguard against them any where. Let every thing be done in the most open manner, let every check be adopted against unfairness, and we do not fear the experiment of resorting to the College.

The evil is a crying one. The pupils of the London Schools have a right to

*demand* the adoption of a remedy. If the malcontents towards the proposed one have a better in their pockets let them bring it out. If they have not, let the students be informed who it is that stands in the way of a supply. If there is not soon a change for the better we shall lay the whole case before our readers and probe its evils to the bottom.

---

### New Regulations of the College of Surgeons.

The palmy days of the London Schools are gone. The competition between themselves, and the growing consequence of the Provincial Schools have already affected the classes, and will affect them more,—

> Which Hebrew priests the more unkindly brook,
> Because the fleece accompanies the flock.

The new Regulations of the College will probably set the seal to the destruction of the private schools, and to the damage of the hospital schools. Not but what the College have been right. It would have been suicidal on its part to hold out against the widely expressed and still more widely felt desire of the Provincial surgeons. Whether the result will be advantageous to the science we will not venture to predict. Whatever renders teaching less profitable will withdraw the best men from it. So far, the result will not be advantageous—but the pupils *may* gain in morals, and *will* gain in purse. How far the latter will operate for the benefit of the profession, let those who already complain of its being overstocked tell us. For our own part we do not hesitate to say that any regulation which operates as a bonus on the entrance of members into the profession, is an evil, and will be felt one.

*Regulations of the Council respecting the professional education of candidates for the diploma.*

I. Candidates will be required, in addition to a Certificate of being not less than twenty-one years of age, to bring proof—

1. Of having been engaged in the acquirement of professional knowledge during a period of not less than four years ; six months of which shall have been occupied in the study of Practical Pharmacy, six months by attendance on the practice of physic, and the remainder of the period on the practice of surgery, at a recognised hospital or hospitals in the united kingdom :—three months being allowed for a vacation in each year.
2. Of having studied anatomy and physiology, by attendance on lectures and demonstrations, and by dissections, during three anatomical seasons or sessions ;*—and of having attended at least two courses of lectures on the principles and practice of surgery, delivered in two distinct periods or seasons, each course comprising not less than 70 lectures :—And one course of not fewer than 70 lectures on each of the following subjects, viz. the practice of physic—chemistry—materia medica—and midwifery, with practical instructions ;

II. Members and licentiates in surgery of any legally constituted college of

---

* An anatomical season is understood to extend from October to April inclusive, and to comprise at least 140 lectures on anatomy and physiology, occupying not less than one hour each, given on separate days ; and at least 100 demonstrations of the like duration, given in a similar manner ; exclusive of dissections, of which distinct certificates are required.

surgeons in the united kingdom, and graduates in surgery of any university requiring residence to obtain degrees, will be admitted for examination on producing their diploma, license, or degree, together with proofs of being 21 years of age, and of having been occupied at least four years in the acquirement of professional knowledge.

III. Graduates in medicine of any legally constituted college or university requiring residence to obtain degrees, will be admitted for examination, on adducing, together with their diploma or degree, proof of having completed the anatomical and surgical education required by the foregoing regulations.

IV. Certificates will not be recognised from any hospital unless the surgeons thereto be one of the legally constituted colleges of surgeons in the united kingdom; nor from any school of medicine or midwifery, unless the respective teachers be members of some legally constituted college of physicians or surgeons in the united kingdom, nor from any school of anatomy or surgery in England, unless the respective teachers be members of some legally constituted college of physicians or surgeons in the united kingdom, and have undergone a second or special examination on those branches of science, according to the ordinances of this college relating thereto.

V. Certificates will not be received on more than one branch of science from one and the same lecturer; but anatomy and physiology—demonstrations and dissections—will be respectively considered as one branch of science.

N.B. In the certificates of attendance on hospital practice and on lectures, it is required that the dates of commencement and termination may be inserted in words at length.

Blank forms of the required certificates may be obtained on application to the secretary, to whom they must be delivered, properly filled up, ten days before the candidate can be admitted to examination; and all such certificates are retained at the college.

By order of the council,
EDMUND BELFOUR, *Sec.*

August 20th, 1839.

